# Intermodal Priming of Cognitive Conflict? A Failed Replication of Mager et al. (2009)

**DOI:** 10.3389/fnhum.2021.680885

**Published:** 2021-06-11

**Authors:** Daniel Wiswede, Jascha Rüsseler

**Affiliations:** ^1^Department of Neurology, University of Lübeck, Lübeck, Germany; ^2^Department of Psychology, Otto-Friedrich-University, Bamberg, Germany

**Keywords:** priming, cognitive conflict, stroop-task, cognitive control, replication

## Abstract

**Introduction:** The present study was conducted to verify a promising experimental setup which demonstrated an inversed Stroop-effect (much faster responses for incongruent relative to congruent Stroop trials) following a mismatching tone. In the matching condition, which was an almost exact replication of the original study, participants were required to indicate whether word color and word meaning were matching, whereas in the response conflict condition, instruction was the same as in a classical Stroop task and required the participants to respond to the word color. As in the original study, each trial was preceded by a sine tone which was deviant in pitch in 20% of the trials.

**Results:** The main result was that the Stroop effect was not inversed after deviant tones, neither under the matching task instruction nor under the response conflict task instruction. The Stroop effect was unaffected by the previous “conceptual mismatch.”

**Conclusion:** The current study failed to replicate the astonishing concept of “conflict priming” reported in previous work and does not open the doors for a new window on sequences of conflicts. Nevertheless, the failed replication is valuable for future research, since it demonstrated that “Conflict Priming” as a facilitation of processing of conflict trials following deviant tones, is not an confirmed finding.

## Introduction

Goal directed behavior requires fast behavioral adaptation to an ever changing environment. This includes the detection of change and the modification of subsequent behavior. Processes involved in monitoring or regulation of strategy, in selecting contextually relevant information and in organizing and optimizing information processing are summarized as “cognitive control processes.”

Stroop- or flanker tasks are often used to examine cognitive control processes. Both paradigms share the same basic logic: Participants are required to give a speeded response to a task-relevant stimulus dimension (a specific letter or sign in the flanker task or a font color in the Stroop task) while ignoring the task-irrelevant dimension, which might either call for the same or for a different response than the central information (congruent or incongruent trials, respectively). Conflict emerges in incongruent trials because the task- relevant and the task-irrelevant information activate different and competing response representations. The presumed role of cognitive control is to enhance the processing of task-relevant stimulus attributes while suppressing task-irrelevant information.

A reliable behavioral indicator of cognitive conflict and for the efficiency of cognitive control is the congruency effect, that is increased reaction times for incongruent relative to congruent trials. Ongoing, dynamic, trial-by-trial changes in cognitive control can be demonstrated by the conflict adaptation effect (for review, see Gratton et al., 1992; Egner, 2007), that is the congruency effect (faster responses for congruent relative to incongruent stimuli) is less pronounced when the current trial was preceded by an incongruent trial than when it was preceded by a congruent trial. One influential model of online adjustment of cognitive control has been proposed by Botvinick and coworkers (conflict monitoring theory; see Botvinick et al., 2001). It states that the anterior cingulate cortex detects conflict and conveys this information to other brain regions, particularly the prefrontal cortex. Here, it is used to adjust cognitive control, resulting in better performance directly after the conflict. However, conflict adaptation can sometimes be explained more parsimoniously by stimulus-response priming instead of conflict-driven adaptations of cognitive control processes. For example, Mayr et al. (2003) found a smaller congruency effect in a standard flanker paradigm after incongruent trials only in stimulus sequences containing exact stimulus-response repetitions, but not in stimulus sequences containing stimulus changes. Thus, the conflict adaptation effect might to some extent be driven by exact stimulus-response repetitions (Nieuwenhuis et al., 2006). However, other research suggest that control-related portions of the effect remain, even if episodic retrieval effects are controlled for (Ullsperger et al., 2005; Egner et al., 2010; Huber-Huber and Ansorge, 2018).

In search for a task to overcome the stimulus-response repetition confound immanent in experiments based on typical Stroop or flanker tasks, we became aware of a study presented by Mager et al. (2009). They used a modified version of the Stroop task, initially developed to disentangle stimulus- and response-related conflicts (Mager et al., 2007, 2009). Participants decided by key press whether there is a match or a mismatch between stimulus color and word meaning (this instruction differs from most other Strop tasks, see below). A task-irrelevant tone was presented prior to each Stroop trial. The tone was either standard (80% of the trials) or deviated in tone pitch (oddball; 20% of the trials). Mager et al. (2009) argued that there is a large overlap between conflict and mismatch on a conceptual level: Conflict is generally understood as a competition of two different stimulus features at the level of response selection or at the level of stimulus representation. Mismatch can also be regarded as a conflict between stimulus representations, namely between the stored representation of the old, standard tone and the incoming representation of the new, deviant tone. Thus, apparently, this experimental design can be understood as an alternative operationalization to study conflicts in close succession: a first conflict, caused by auditory mismatch, is succeeded by a conceptual conflict caused by the Stroop- like stimulus. On the behavioral level, they found the default congruency effect for Stroop stimuli following standard tones. Most interestingly, the congruency effect was reversed after deviant tones; participants were faster on incongruent compared to congruent Stroop stimuli following deviant tones. The authors concluded that deviant auditory stimulation improves processing of subsequent conflicts and labeled this cross-modal effect “conflict priming.”

This kind of “conflict priming” cannot be explained simply by assuming orientation of attention toward the stimulus, since this should affect reaction time for matching and non-matching Stroop stimuli in the same manner. Since the tones did not require a response and there are no similarities between auditory and visual stimuli in this task, the stimulus-response-repetition confound immanent in flanker-based conflict adaptation effects cannot account for the results either. Mager et al. (2009) state “… the first deviance-related mismatch may prepare the conflict-related brain structures for more efficient conflict processing and resolution of the subsequent visual conflict” (p. 2186/87). Thus, although the authors did not explicitly link conflict priming to the conflict monitoring theory, the basic idea is similar and the data might be explained within the conflict monitoring framework: A deviant auditory tone signals the occurrence of conflict, which prompts the cognitive system to increase cognitive control. This increase in cognitive control facilitates detection and resolution of the subsequent conflict, indicated by decreased reaction times in high-conflict Stroop trials following mismatching tones. Results provided by Mager et al. (2009) allow also calculation of the conflict adaptation effect following the reasoning outlined in Nieuwenhuis et al. (2006) by subtracting the congruency effect (RT incongruent trials-RT congruent trials) in high conflict (=deviant tones) from the congruency effect in low conflict trials (=standard tones) (731–690 ms)—(693–721 ms). This results in an astonishing large conflict adaptation effect of 69 ms. In addition, the idea of conflict priming seems highly promising to us, since it is in contrast to previous research showing that deviant and novel sounds prolong reaction times (Escera et al., 2001), whereas Mager and colleagues report general reaction time decrease after tone deviance.

Taken together, the concept of conflict priming might provide a valuable contribution to our understanding of cognitive control without confounds related to exact stimulus-response-repetitions. Thus, it seems promising to establish the cross-modal priming paradigm developed by Mager et al. for future research. However, since previous research has shown that also the field of (behavioral) neuroscience suffers from low reproducibility (Button et al., 2013), the purpose of the present studies was to provide an independent replication and extension of Mager et al. (2009) behavioral findings. A task-irrelevant deviant or standard tone was followed by a visual stimulus containing some kind of conflict. To keep comparability with Mager et al., one part of the experiment was conducted with a matching instruction, resulting in an (almost) direct replication of the Mager et al. (2009) study. However, this kind of “matching instruction” is somewhat unusual for a Stroop task, in which participants are normally required to respond to one stimulus dimension, in most cases by naming the color of the stimulus. To examine whether conflict priming is also apparent in a classical Stroop task, in which participants are facing a response conflict between font color and word meaning, another part of the experiment employed a standard response conflict version of the Stroop task.

## Materials and Methods

### Power Calculation and Participants

**Figure 1:**
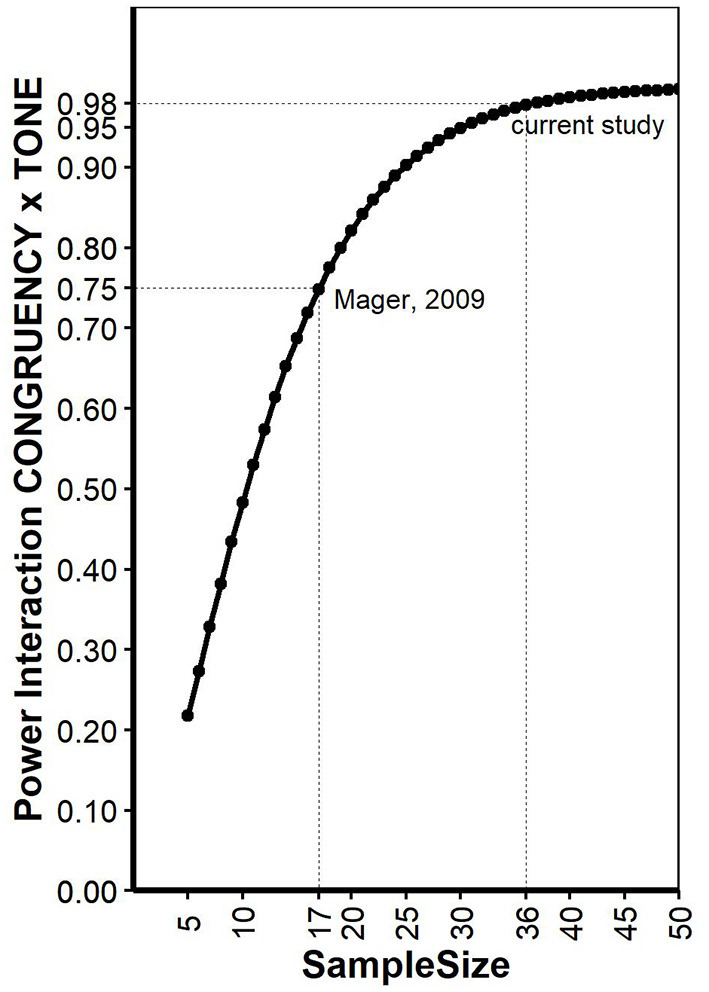
Power calculations for different sample sizes for the interaction “CONGRUENCY x TONE.” See text for explanations.

As pointed out earlier (Potvin and Schutz, 2000), determining a priory power for repeated measurement ANOVA is not trivial, since quantifying the error variance is tricky. Here, we conducted power analysis for a 2 × 2 within-within design ANOVA as described for the reaction times in Mager et al. (2009) on a simulation-based approach implemented in the R package “Superpower” (Caldwell and Lakens, 2019). The function “ANOVA_exact” simulates an exact dataset that matches the desired properties reported in Mager et al. (2009) and provides power for the interaction effect. Mean values of all conditions were provided in Mager et al. (2009): standard deviations for each condition was estimated based on standard errors given in Figure 1 and number of participants and set to 111.3, 101.8, 101.8, and 105.6 for the factors CONGRUENCY (con, inc) and TONE (standard, deviant), respectively. Reliability for a Stroop task was estimated based on literature (Franzen et al., 1987) and set to 0.77. Alpha level was set to 0.05. The same analysis was also conducted using the simulation function “ANOVA_power,” a R package to simulate factorial designs and empirically calculate observed power for interactions in a ANOVA based on 10,000 simulations. This analysis yielded very similar results with the same conclusions. The power of Mager et al. (2009) interaction was estimated with 0.75. The same calculations were repeated for increasing sample sizes, allowing to estimate a sample size for the current study with setting type 2 error = type 1 error ≤ 0.05. As pointed out elsewhere (Lakens, 2013), Power of 0.80 is the recommended minimum, but higher power (e.g., 0.95) is more desirable. Our analysis revealed that Power of 0.95 can be expected if data of at least 30 participants are collected. For the current study, we decided to collect data of 30 participants with additional 20% to decrease the danger of data loss due to unexpected error rates or other indications of low compliance. Thus, data were collected from 36 participants (19 women, range: 19-28 years), and none of the data sets had to be excluded. All participants reported normal or corrected to normal vision and no known hearing problems. The experiment lasted around 45 min, participants were reimbursed with four Euro and a chocolate. The study conforms with The Code of Ethics of the World Medical Association (Declaration of Helsinki), printed in the British Medical Journal (18 July 1964).

#### Material, Design, and Procedure

A trial started with auditory presentation of a standard or deviant tone for 100 ms (80 or 20% probability, 450 Hz or 550 Hz, respectively, same as in Mager et al., 2009). Three hundred milliseconds after tone onset, participants were presented with a visual stimulus, consisting of the (German) words and colors red, green, blue, and yellow (rot, grün, blau, and gelb) on black background.

There were two instructions: In the “Matching task” instruction, participants were required to decide whether word color matches or did not match word meaning (=congruent and incongruent trials, respectively). This task is equivalent to the modified Stroop task reported in Mager et al. (2009). Responses were given with both index fingers. For half of the participants, the “match” button was mapped to the right index finger, for the others it was mapped to the left index finger. For half of the Stroop stimuli, the font color matched the color name. As in the original study, the order of the stimuli was pseudorandomized in that there were never more than three identical colors or words and no more than four identical congruency levels. In the “response conflict” instruction, participants had to report the word color using a special purpose four buttons response device. This task was designed to mirror a typical Stroop instruction. One quarter of the Stroop stimuli was congruent in that the font color matched the word meaning, the remaining three quarters were incongruent. Responses were given with the index and middle fingers of both hands, a colored sticker on each button indicated the color allocation. During the experiment, participant's fingers covered the colored dots so that they could not see it any longer. Trials were presented randomly with the restriction that (a) no more than three repetitions of the same word or same color and (b) a maximum of four matches or mismatches in a sequence.

Under both instructions, the congruent and incongruent visual stimuli were equally distributed with respect to the preceding tone. Button press or no response for 1,000 ms terminated Stroop stimulus presentation; the time between two Stroop stimuli varied randomly between 2050 and 2150 ms (same as in Mager et al., 2009).

The complete experiment with both instructions consisted of eight blocks with 120 trials each, separated by a short break. The “matching” or “response conflict” instruction was either provided at the beginning of the experiment or after four blocks; the sequence of instructions (matching first vs. response conflict first) was counterbalanced across participants. A task instruction was provided at the beginning of the experiment and prior to instruction change. After instructions, participants received 40 practice trials, feedback for slow responses (>800 ms) or errors was provided. If participants responded too slow (>800 ms) or erroneously in more than 20 percent of the trials, another practice block was administered. As in Mager et al. (2009), participants were also instructed to ignore the tones and respond only to the visual stimuli.

The experiment was conducted in a laboratory environment, which allows to test eight participants simultaneously on standard IBM PCs which were separated by core walls. Participants were placed with eye distance of 75 cm from the middle of a standard monitor, stimuli were presented in Courier font size 16. Responses were given on a special purpose response device. Tones were presented via headphones; volume was tested prior to the experiment by three student assistants so that the tones appear loud, but not painful. Volume was kept constant across all participants. The experiment was programmed and presented using Eprime 2.0.

To summarize, subjects had to match both stimulus dimensions [matching task instruction, replication of Mager et al. (2009)] or had to react to one stimulus dimension (response conflict instruction). Each conflict stimulus was preceded by either a standard or a deviant tone. Deviant tones were presented with the same probability prior to congruent and incongruent conflict stimuli.

#### Data Analysis

We conducted a within subjects Analysis of Variance (ANOVA) with factors TASK (two levels: matching task vs. response conflict task), CONGRUENCY (two levels; congruent or incongruent Stroop stimuli) and TONE (two levels, standard or deviant tone prior to Stroop stimulus). Prior to data analysis, 0.73% of the trials were excluded, most of them (0.7%) because of no responses within 1,000 ms after Stroop stimulus onset, the remaining because of response times faster than 200 ms. All calculations and figures were conducted with R (R Core Team, 2016), all figures were generated with ggplot2 (Wickham, 2009). ANOVA Statistics were conducted with the R package EZanova (Lawrence, 2016; R Core Team, 2016). Raw data are publicly available (http://dx.doi.org/10.23668/psycharchives.4824).

## Results

### General Task Effects

Participants were faster in the response conflict task (504 ms) than in the matching task [537 ms, *F*_(1, 35)_ = 15.8; *p* < 0.01]. There was the tendency that the congruency effect was more pronounced in the matching task (difference incongruent minus congruent = 43 ms) than in the response conflict task (difference incongruent minus congruent = 34 ms, interaction TASK × CONGRUENCY, *F*_(1, 35)_ = 3.21; *p* < 0.08]. There were no significant TASK x TONE nor TASK × CONGRUENCY × TONE interaction. Effects of congruency and tone will now be reported in more detail separately for both tasks.

### Matching Task

Faster responses were given when the word color matched the word meaning [Stimulus Conflict: RTs congruent 515 ms, incongruent 558 ms; *F*_(1, 35)_ = 74.4; *p* < 0.01, partial Eta = 0.68]. Mismatching tones had no general effect on response speed [TONE n.s.; standard tone: 536 ms, deviant tone 537 ms, *F*_(1, 35)_ < 1]. Deviant tones did not decrease reaction times for incongruent stimuli; nor did tones influence congruency effects in any other way [TONE × CONGRUENCY interaction, *F*_(1, 35)_ = 2.7; *p* < 0.11, congruency effect after standard tones: 40 ms; congruency after deviant tones: 46 ms, partial Eta = 0.07]. Thus, in contrast to Mager et al. (2009), the congruency effect was numerically somewhat larger, but not significantly smaller after deviant tones. The pure conflict adaptation effect (congruency effect in standard tone trials minus congruency effect in deviant tone trials) was −6 ms. Thus, there was no conflict reduction after high conflict at all; we could not replicate the immense conflict adaptation effect of +69 ms found in Mager et al. (2009). Error rates were only influenced by stimulus congruency with less errors for congruent relative to incongruent trials [10 vs. 12% errors, *F*_(1, 35)_ = 4.61; *p* < 0.04; TONE or interaction n.s.]. To estimate conflict adaptation on an individual level, the congruency effect was plotted separately for each participant (see **Figure 3**). Given the matching instruction, the “Conflict Priming” pattern as reported in Mager et al. (2009) was found in one participant. A “normal” congruency effect (Δ RT incongruent—congruent >0) was found in 33 out of 36 participants. Two participants showed an unclassified pattern with inversed congruency effect for deviant and standard tones. Neither the participant with conflict priming pattern nor both participants with unclassified pattern showed extreme values in general RTs or error rates. Thus, to find a “conflict priming” pattern was not more likely than to find a generally inversed congruency effect; even “mild” conflict priming with a speed advantage following deviant relative to standard tones without inversion of the congruency effect was not more likely than no conflict priming. For the response instruction, a “Conflict Priming” pattern was shown in 4 out of 36 participants; none of them showed conflict priming under the matching instruction.

### Response Conflict Task

**Figure 2:**
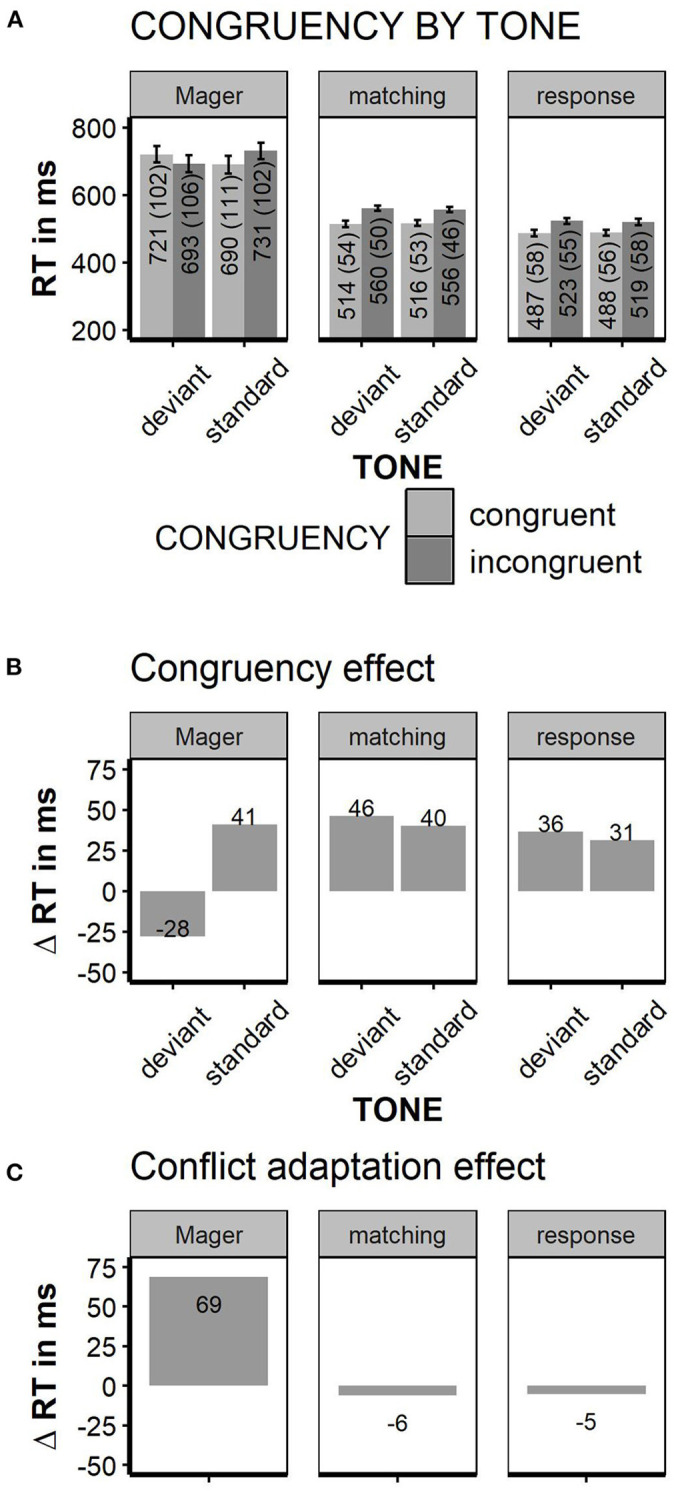
Reaction times. Left columns data from Mager et al. (2009), middle and right column matching task instruction replication of Mager et al. (2009) and response conflict instruction. **(A)** Reaction times grouped by factors “TONE” and “CONGRUENCY.” **(B)** Congruency effect, obtained by subtracting RT for congruent from the incongruent trials. **(C)** “conflict adaptation effect,” obtained by subtracting the congruency effect in high conflict trials (deviant tones) from the congruency effect in low conflict trials (= standard tones); error bars indicate ±1 SE; error bars for Mager are estimated based on Figure 1 in Mager et al. (2009).

Although the responses were generally faster and less error-prone than in the Matching task (537 ms matching task vs. 504 ms response conflict task; 12.9 vs. 7.2% errors, respectively), the overall RT pattern was quite similar in both instruction conditions. Again, participants responded faster to congruent relative to incongruent trials [487 vs. 521 ms, *F*_(1, 35)_ = 132.3; *p* < 0.01, partial Eta = 0.79]. Deviant tones did neither influence overall RTs [RT, standard tone trials = 504 ms, deviant tone trials = 505 ms, *F*_(1, 35)_ < 1] nor did they interact with stimulus congruency [TONE × CONGRUENCY, *F*_(1, 35)_ < 1, partial Eta 0.02]. The pure conflict adaptation effect, calculated as above, was −5 ms. The overall error rate was neither influenced by congruency nor tone (error rates; congruent vs. incongruent trials: 6.5 vs. 6.5%; standard tone vs. deviant tone trials: 6.5 vs. 6.4%, all main effects and interaction n.s.). See Figure 2 for summary of all reaction times.

## Discussion

**Figure 3:**
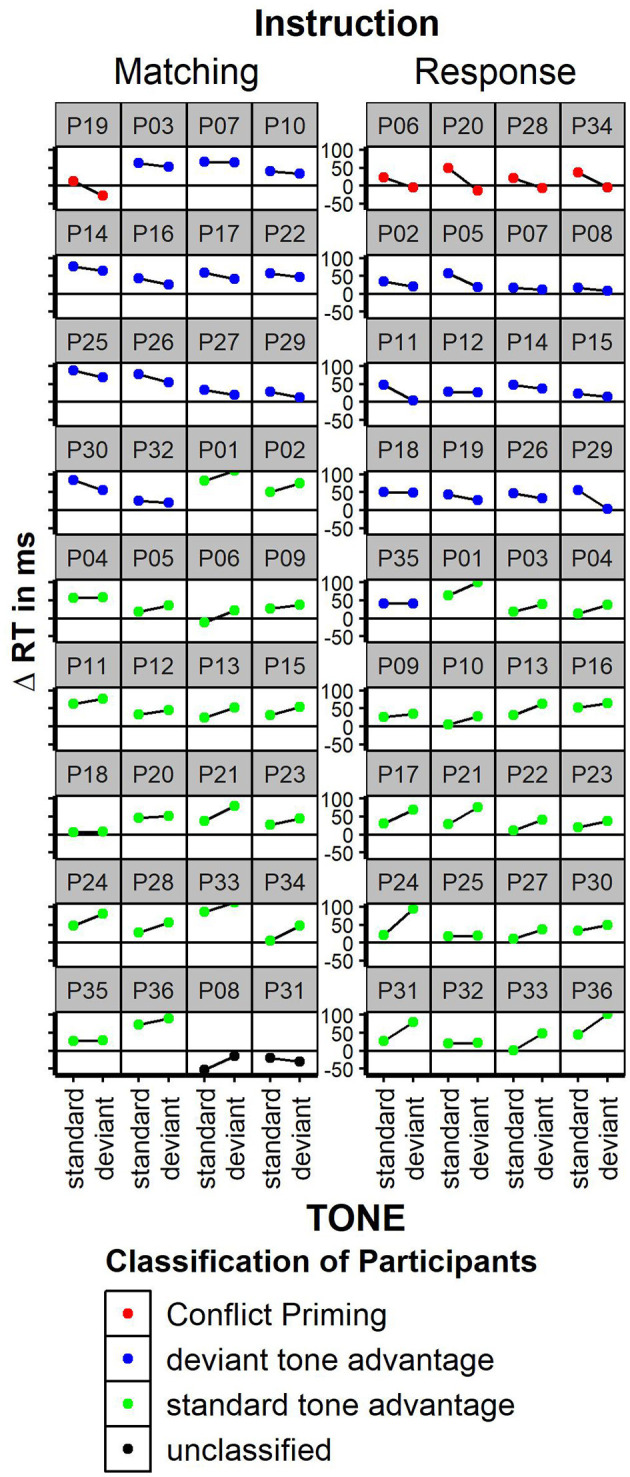
Congruency effect (incongruent—congruent trials) on the individual participant level for the matching instruction and response instruction. Participants were classified based on the congruency effect. “Conflict priming” = positive congruency effect following standard tones and negative congruency effect following deviant tones. This replicates the pattern reported in Mager et al. (2009). “deviant tone advantage” = smaller congruency effect following deviant tones. “standard tone advantage”: smaller congruency effect following standard tones. “unclassified”: unusual congruency effect with inverted congruency effects. Facet figures are sorted by congruency classification.

The experiment reported here explored intermodal priming of cognitive conflict. Participants performed two modified versions of a Stroop-task. They had to decide whether the target and the distractor stimuli were identical (matching task instruction) or they had to respond to the identity of the target stimulus (response conflict task instruction). A task-irrelevant tone was presented prior to each visual stimulation, which was deviant in tone pitch in 20% of the trials. This manipulation was introduced to study the intermodal priming of cognitive conflict introduced by Mager et al. (2009). These authors reported that the congruency effect is reversed after the presentation of deviant tones, that is responses to incongruent trials were faster compared to responses to congruent trials. In the present study these findings could not be replicated: the congruency-effect was of the same magnitude regardless of the presentation of the tone, that is the congruency-effect was not different after the presentation of a standard or a deviant irrelevant tone. Thus, in contrast to the previously reported findings, conflict priming was completely absent in the present study. As the present replication has sufficient power to detect an effect of the size of that reported by Mager et al. (2009), we conclude that it is an unsolved question whether priming of cognitive conflict exists. As seen by plotting the congruency effect on the individual level (Figure 3), conflict priming might occur, but it is a pattern within the normal variation of behavior. In the five cases with conflict priming (only one in the Mager-replication), the inversed congruency effect following deviant tones was not associated with unusual patterns of reaction times, conflict adaptation or error rates. Ultimately, we cannot offer an explanation as to why conflict priming could not be replicated. However, it looks as if there were elementary differences in the execution of the task or the evaluation of the data, which is indicated by the significantly slower reaction times in Mager et al. (2009) (see Figure 2A). It is also not reasonable to assume that differences in outlier analysis can account for our null finding. We also reanalyzed the data without excluding any outliers and with more sophisticated outlier analysis methods (i.e., using Tukey's Hinges Tukey, 1977), which did not change the general data pattern.

Similar to our current work, Rünger et al. (2010) failed to replicate earlier findings by Fernandez-Duque and Knight (2008, Experiment 4). These authors reported an across-task effect of endogenously generated, anticipatory control: In their study, a cue that predicted conflict in an upcoming Eriksen flanker task modulated conflict regulation in a subsequent number Stroop task. Thus, it seems that the intermodal priming of cognitive conflict is a finding that may, if at all, be present only under certain experimental conditions that have to be uncovered by future experimentation.

Replications of important research findings gain importance in psychology and in neuroscience (Button et al., 2013; Baxter and Burwell, 2017). Although the paper by Mager et al. (2009) has only been cited seven times, the issue of adaptive control processes in tasks eliciting cognitive conflict is actively investigated by several research groups (e.g., Schuch and Koch, 2015; Huber-Huber and Ansorge, 2018; Berger et al., 2019) and several paradigms have been used to investigate this topic. The conflict priming paradigm introduced by Mager et al. (2009) provides a potentially interesting paradigm to study cognitive control processes as it avoids stimulus overlap in succeeding trials. Thus, it is important to establish the reliability of the reported effect of priming of cognitive conflict. Unfortunately, the present study shows that the paradigm developed by Mager et al. (2009) does not reliably elicit priming of cognitive conflict and, thus, cannot be used to study cognitive control processes.

## Data Availability Statement

The datasets for this study can be found in the Psycharchives http://dx.doi.org/10.23668/psycharchives.4824.

## Ethics Statement

Ethical review and approval was not required for the study on human participants in accordance with the local legislation and institutional requirements. The patients/participants provided their written informed consent to participate in this study.

## Author Contributions

DW and JR designed the study and wrote the paper. DW collected and analyzed the data. All authors contributed to the article and approved the submitted version.

## Conflict of Interest

The authors declare that the research was conducted in the absence of any commercial or financial relationships that could be construed as a potential conflict of interest.
